# Investigation of the Immune Modulatory Potential of Zinc Oxide Nanoparticles in Human Lymphocytes

**DOI:** 10.3390/nano11030629

**Published:** 2021-03-03

**Authors:** Helena Moratin, Pascal Ickrath, Agmal Scherzad, Till Jasper Meyer, Sebastian Naczenski, Rudolf Hagen, Stephan Hackenberg

**Affiliations:** Department of Oto-Rhino-Laryngology, Plastic, Aesthetic and Reconstructive Head and Neck Surgery, University of Wuerzburg, 97080 Wuerzburg, Germany; scherzad_a@ukw.de (A.S.); meyer_t2@ukw.de (T.J.M.); sebastian3004@t-online.de (S.N.); hagen_r@ukw.de (R.H.); hackenberg_s@ukw.de (S.H.)

**Keywords:** zinc oxide nanoparticles, immunomodulation, T cell subpopulations

## Abstract

Zinc oxide nanoparticles (ZnO-NP) are commonly used for a variety of applications in everyday life. In addition, due to its versatility, nanotechnology supports promising approaches in the medical sector. NP can act as drug-carriers in the context of targeted chemo- or immunotherapy, and might also exhibit autonomous immune-modulatory characteristics. Knowledge of potential immunosuppressive or stimulating effects of NP is indispensable for the safety of consumers as well as patients. In this study, primary human peripheral blood lymphocytes of 9 donors were treated with different sub-cytotoxic concentrations of ZnO-NP for the duration of 1, 2, or 3 days. Flow cytometry was performed to investigate changes in the activation profile and the proportion of T cell subpopulations. ZnO-NP applied in this study did not induce any significant alterations in the examined markers, indicating their lack of impairment in terms of immune modulation. However, physicochemical characteristics exert a major influence on NP-associated bioactivity. To allow a precise simulation of the complex molecular processes of immune modulation, a physiological model including the different components of an immune response is needed.

## 1. Introduction

Nanotechnology is a rapidly evolving field and is considered one of the key technologies of the 21st century. The extent of this multidisciplinary technological branch ranges from daily consumer products in the food and cosmetics industry to advanced medical applications [1]. To be defined as a nanomaterial (NM), at least 50% of the components must measure between 1 and 100 nm. Nanoparticles (NP) exhibit their nanoscale in three dimensions [2]. The term nanomedicine has recently emerged to describe the implementation of NM in the medical sector. It implies the usage of NM in prevention, diagnostics and therapy of various diseases. Due to their extremely small size, and thus increased physicochemical reactivity, NP can overcome barriers that normally represent physiological limitations for other common therapeutic substances. Nanotheranostics refers to substances that can function as therapeutics and diagnostic imaging agents. Nanomaterials such as carbon quantum dots, for example, can improve the radiological discrimination between conspicuous and healthy tissue, or act as nano-carriers for a targeted drug therapy [3,4,5]. In this context, NP with specific antigen-compatible surface modifications can be used for the delivery of small drug molecules, as well as biomolecules such as DNA, RNA or proteins to the target site [6]. These properties are not only particularly interesting for oncological therapies, but also for the concise induction of bioactivity in the organism, for example in connection with vaccination.

Metal oxides like titanium dioxide (TiO_2_) and zinc oxide are among the most frequently used substances for NM production in general [7]. ZnO has a variety of unique physicochemical properties including a wide range of radiation absorption and high photostability, which makes it an ideal ingredient for sunscreen products [8]. Furthermore, it is commonly used for food packaging due to its antimicrobial effects. Its most frequent application is for the production of various cosmetics including toothpaste and skin care products. In addition, the use of ZnO in the medical field is very promising due to its anti-inflammatory, antimicrobial and antifungal potential, for example in the development of special textiles for patients with chronic inflammatory skin diseases [9]. Moreover, ZnO-NP bound with chemotherapy drugs showed selective anti-cancer activity in vitro [10].

However, there is still a lack of precise data on the potential risk factors associated with NP exposure. Particularly workers in the chemical industry who are involved in the production of NP might have repetitive low-level contact, which should be considered carefully in this context. For example, translocation of NP to peripheral organs, including lung-associated lymph nodes, has been previously observed [11], plus cytotoxicity and genotoxicity have also been evaluated in multiple studies [12,13,14]. However, the modification of the immunoresponse has not yet been the focus of adequate investigation when considering the potential consequences ranging from allergic to systemic inflammatory reactions.

Increasing exposure of humans to NM in everyday life, as well as in the context of medical care, must raise awareness as to the possible interactions with nanoscale structures in the human organism. Precise knowledge of the effects of NM on the immune response is critical for a variety of different reasons. On the one hand, particularly in the context of an application in drug therapy, adverse immune-related toxicity can have extensive consequences for patients. On the other hand, understanding the NM-associated immunomodulation can help improve the efficacy of vaccines or immunotherapies. This study was designed to obtain constructive information on the potential of ZnO-NP to induce or impede the activation of immune cells. To this end, a broad panel of surface markers indicating cell activation and differentiation of T cell subtypes was applied after incubation of lymphocytes to NP for determined time periods.

## 2. Materials and Methods

### 2.1. Preparation and Characterization of Zinc Oxide Nanoparticles

An amount of 20 mg ZnO-NPs (mknano, Mississauga, ON, Canada) were suspended in 1740 µL distillated aqua and sonicated for 120 sec (Bandelin, Sonopuls HD 60, Berlin, Germany) for particle dispersion. Then, 60 µL of 1.5 mg/mL bovine serum albumin (BSA) and 200 µL 10-fold concentrated phosphate buffered saline (PBS) were added to create a stable and physiological stock suspension. This stock suspension was diluted with PBS to achieve the required exposure concentrations.

The precise process of particle characterization has been described previously [15]. To evaluate size, shape, and aggregation tendency, transmission electron microscopy (TEM) was performed on a Zeiss transmission electron microscope EM 900 (Carl Zeiss, Oberkochen, Germany) at the Division of Electron Microscopy at the University of Wuerzburg Biocenter. TEM samples were prepared by drop coating the stock suspension on carbon-coated copper grids after sonication and stabilization. The films on the grids were dried using a tissue paper before measurement. Zeta potential and size distribution of NP aggregates were measured by dynamic light scattering (Malvern Instruments Ltd., Herrenberg, Germany). 

### 2.2. Preparation of Human Lymphocytes

Human lymphocytes were obtained from nine healthy donors after obtaining informed consent according to approval of the Medical Department Ethics Board of the Julius Maximilians University Wuerzburg (116/17). Heparinized blood was processed by density-gradient centrifugation (10 min, 1.000× *g*) in a membrane containing 10 mL cell tube (Greiner Bio-One, Frickenhausen, Germany) at room temperature (RT) on equal amounts of Ficoll (Biochrom, Berlin, Germany) in order to separate the constituents. The lymphocyte fraction was carefully collected and washed twice in PBS. Cell viability and number were then determined by electronic cell counting (Casy Technology, Innovatis AG, Reutlingen, Germany). 

### 2.3. Annexin-V/Propidiumiodide Fluorescence-Activated Cell Sorting (FACS)

To determine an appropriate sub-cytotoxic concentration range for the further flow cytometric measurements, Annexin-V/Propidiumiodid FACS was performed according to the manufacturer’s protocol (Becton-Dickinson Bioscience, Heidelberg, Germany). After electronic cell counting, 2 × 10^5^ cells were treated with ZnO-NP concentrations between 0.1 and 100 µg/mL for 24 h. Here, the stock suspension of NP was diluted with PBS to achieve the right NP concentration. The particles were then added to the cell culture medium. After exposure, cells were washed with PBS twice before resuspending the cell pellet with 100 µL binding buffer. 5 µL Annexin V-APC and 5 µL Propidiumiodide were then added to stain the cells. Fluorescence was measured by flow cytometry (FACScanto, Becton-Dickinson). 

### 2.4. Flow Cytometry Analysis

The following antibodies were used: Anti-CD45 Pacific Orange (Thermo Fisher Scientific, Inc., Waltham, MA, USA), anti-CD3 phycoerythrin-Cyanine 7 (PE.Cy7), anti-CD4 Pacific Blue, anti-CD8a Alexa 700, anti-CD45RA peridinin chlorophyll protein complex-Cy5.5 (Per.CP-Cy5.5), anti-CD4 fluorescein isothiocyanate (FITC), anti-Forkhead-Box-Protein P3 (FoxP3) Pacific Blue, anti-cytotoxic T-lymphocyte-associated Protein 4 (CTLA-4) R-Phycoerythrin (PE), anti-human leukocyte antigen (HLA)-DR isotype AlexaFluor 700, anti CD69 allophycocyanin (APC), anti-CD38 Per.CP-Cy5.5, anti-CCR7 Alexa488, Anti-CD25 APC (all from BioLegend, Inc., San Diego, CA, USA) and anti-Ki-67 (BD Biosciences, San Jose, CA, USA). Isotype control staining was performed using mouse-immunoglobulin G (IgG) antigen-presenting cell (APC; BioLegend, Inc.) and mouse-IgG PE (BD Biosciences). Viability Dye 780 (eBioscience; Thermo Fisher Scientific Inc.) was used to detect apoptotic and dead cells. After blocking with 25 μg/mL normal mouse IgG (Sigma-Aldrich; Merck KGaA, St. Louis, MO, USA) for 15 min on ice, all cells underwent cell surface staining on ice for 30 min, followed by intracellular staining. Flow cytometry analysis was performed as previously described [16,17]. All antibodies were used according to the manufacturer’s protocol. FACS analysis was performed using an LSR II flow cytometer and the data were analyzed using FlowJo 10.2 software (FlowJo LLC, Ashland, OR, USA).

### 2.5. Statistics

Data are presented as the mean ± standard deviation. The statistical significance of data was determined by multiple and unpaired t-tests using GraphPad Prism software 8 (GraphPad Software, Inc., La Jolla, CA, USA). A *p*-value < 0.05 was considered statistically significant.

## 3. Results

### 3.1. Nanoparticle Characterization

The ZnO-NP used in this study were spherical in shape with a mean diameter of –30 nm, according to the manufacturer’s specification. A zeta potential of −11.2 mV and a mean diameter of particle aggregates of 67.06 nm in culture medium was determined by dynamic light scattering.

### 3.2. Annexin-V/Propidiumiodide FACS

**Figure 1:**
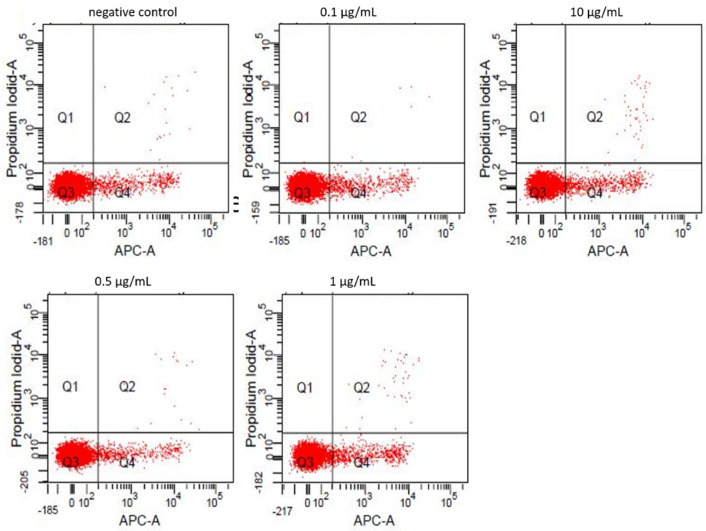
Results of flow cytometric analysis with Annexin-V/Propidiumiodide staining of lymphocytes after treatment with 0.1, 0.5, 1, and 10 µg/mL ZnO-NP for 24 h. The upper left dot-plot shows the untreated reference. Q3 denotes viable cells, Q4 early apoptotic and Q2 late apoptotic/necrotic cells. Data were obtained in one exemplary experiment. There is no notable change in the proportion of viable/apoptotic/necrotic cells after treatment with NP compared to the negative control. APC-A: Allophyocyanin-Annexin-V.

Fractions of viable (quadrant Q3 in dot-plot graph), apoptotic (Q4), and necrotic (Q2) cells were measured after Annexin-V/Propidiumiodide staining by flow cytometry. Untreated cells showed a viability rate of 94.2% (Q3: 5.6%, Q4: 0.2%). Cell viability was not relevantly altered by incubation with ZnO-NP concentrations up to 10 µl/mL (93.7%) (Figure 1). Higher concentrations induced significant dose-dependent apoptosis in lymphocytes. The individual percentages of viable, apoptotic and necrotic cells after NP-exposure in accordance with the dosage are illustrated in Table 1.

### 3.3. Cell Viability, T Cell Subtypes and T Cell Activation

**Figure 2:**
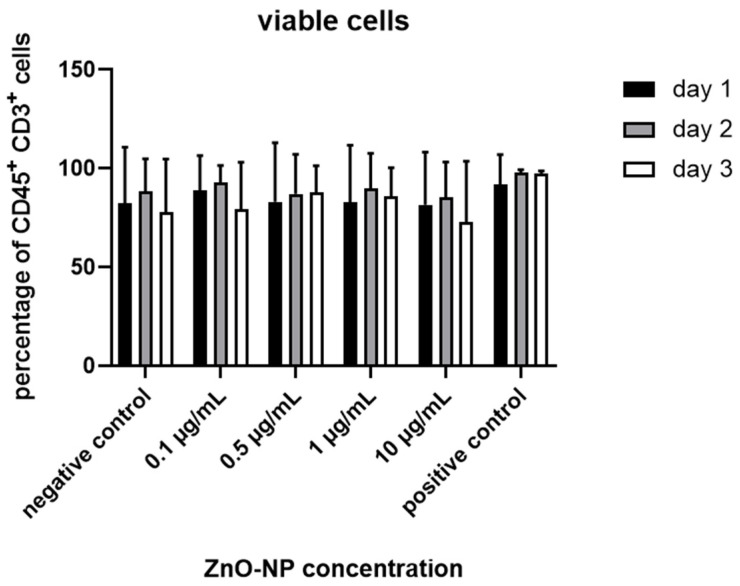
Ratio of viable lymphocytes after incubation with ZnO-NP in different concentrations (0.1, 0.5, 1, 10 µg/mL) for 24, 48, or 72 h. Differences compared to untreated cells (negative control) were not statistically significant. Data represent the mean +/− standard deviation (n = 9 for day 1 and 2, n = 7 for day 3).

**Figure 3:**
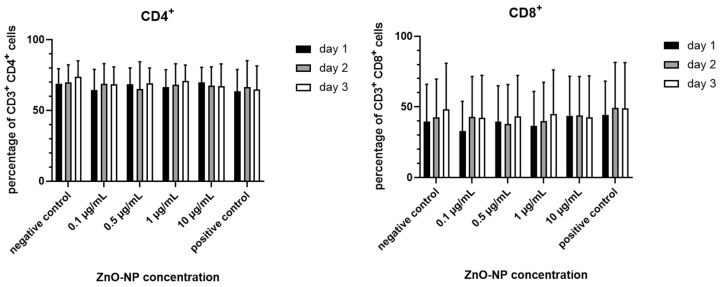
Ratio of CD8^+^ and CD4^+^ T cells after incubation with ZnO-NP in different concentrations for 24, 48, or 72 h. Changes were not statistically significant compared to the negative control. Data represent the mean +/− standard deviation (n = 9 for day 1 and 2, n = 7 for day 3 for CD4^+^ cells; n = 7 for day 1, n = 6 for day 2 and 3 for CD 8^+^ cells).

The proportion of viable CD45^+^ CD3^+^ lymphocytes was not significantly altered after NP-incubation with 0.1, 0.5, 1 and 10 µg/mL even after 3 days (Figure 2). Furthermore, the ratio of CD3^+^ CD4^+^ and CD 3^+^ CD8^+^ T cells remained unaffected by incubation time and dosage (Figure 3). 

**Figure 4:**
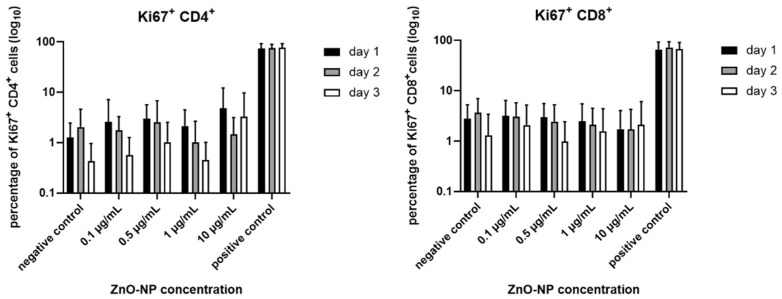
Ratio of Ki67^+^ in CD4^+^ and CD8^+^ T cells. There is a tendency towards a decrease in T cell activation over time. Data are plotted logarithmically, there is no statistical significance in terms of dose and time dependence. Data represent the mean +/− standard deviation (n = 9 for day 1 and 2, n = 7 for day 3 for CD4^+^ cells; n = 7 for day 1, n = 6 for day 2 and 3 for CD 8^+^ cells).

T cell activation was measured by Ki67 expression of CD4^+^ and CD8^+^ T cells. There was a tendency towards a decrease in Ki67 expression in CD4^+^ and CD8^+^ T cells with increasing exposure time, and these changes were independent of NP exposition. Alterations were not statistically significant in terms of time and NP-dosage (Figure 4). 

**Figure 5:**
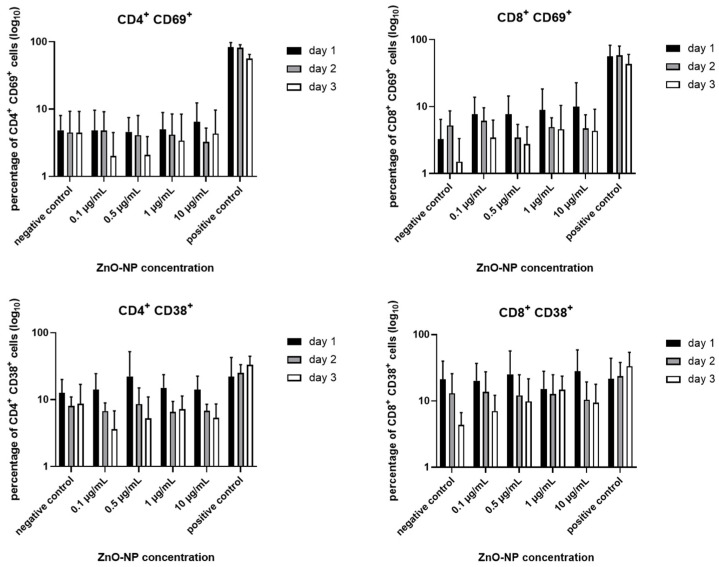
Expression of CD69 and CD38 in CD4^+^ and CD8^+^ T cells. The expression tends to decrease over time, although changes are not statistically significant. Results are presented logarithmically. Data represent the mean +/− standard deviation (n = 9 for day 1 and 2, n = 7 for day 3 for CD4^+^ cells; n = 7 for day 1, n = 6 for day 2 and 3 for CD 8^+^ cells).

According to the Ki67 expression, the proportions of the activation markers CD69 and CD38 showed a decreasing tendency over time on CD4^+^ and CD8^+^ T cells, but no statistical significance (Figure 5).

### 3.4. CD4^+^ T Cell Differentiation and HLA-DR Expression

**Figure 6:**
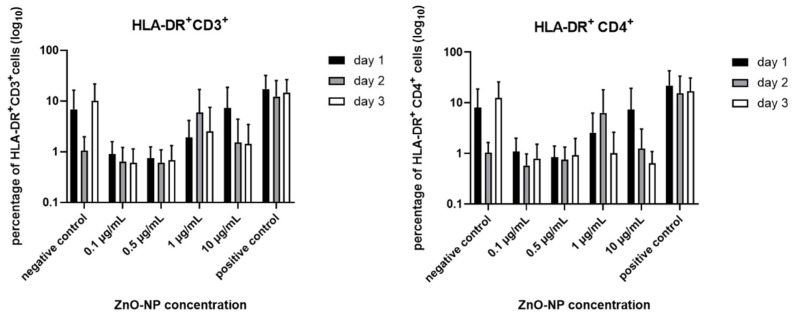
HLA-DR expression in the overall population and in the CD4^+^ subset. The expression declined independent of time in the samples treated with 0.1 and 0.5 µg/mL ZnO-NP, and increased after incubation with 1 and 10 µg/mL, especially on the first treatment day. Data represent the mean +/− standard deviation (n = 9 for day 1, 2, and 3 for CD 3^+^ cells; n = 9 for day 1 and 2, n = 8 for day 3 for CD4^+^ cells).

Expression of HLA-DR was analyzed on CD3^+^ lymphocytes and in the CD4^+^ T cell subset. Figure 6 shows that the measurable expression declined independent of time in the samples which were treated with 0.1 and 0.5 µg/mL ZnO-NP, and increased after incubation with 1 and 10 µg/mL in both groups. These observations were particularly noticeable on the first and second day of NP treatment. 

The influence of NP exposure on CD4+ T cell differentiation was evaluated by analyzing the occurrence of CD3^+^ CD4^+^ CD45RA^+^ FoxP3^–^ naïve and CD3^+^ CD4^+^ CD45RA^–^ FoxP3^–^ memory CD4^+^ cells, CD3^+^ CD4^+^ CD45RA^+^ FoxP3^low^ resting regulatory T cells (rTreg), CD3^+^ CD4^+^ CD45RA^–^ FoxP3^high^ activated Treg (aTreg) and CD3^+^ CD4^+^ CD45RA^–^ FoxP3^low^ memory T cell expression. Table 2, Table 3 and Table 4 show the data of the subtype analysis. The proportion of naïve T cells decreased in a time-dependent manner, while the percentage of memory T cells increased accordingly. NP exposure showed no noticeable influence. Furthermore, the percentage of rTreg and aTreg decreased slightly over time, while FoxP3^low^ memory T cell expression remained stable. These variances were not statistically significant.

### 3.5. Proportion of Terminally Differentiated CD4+ and CD8+ T Cells

**Figure 7:**
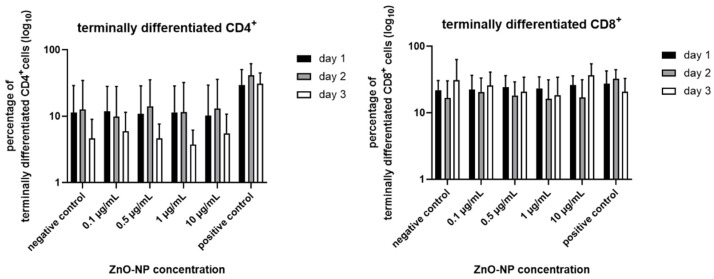
Ratio of terminally differentiated cells in CD4^+^ and CD8^+^ subpopulations. Proportions appear to decline over time but remain unaffected by NP incubation. Alterations are not statistically significant. Data represent the mean +/− standard deviation (n = 9 for day 1 and 2, n = 7 for day 3 for CD4^+^ cells; n = 6 for day 1 and 2, n = 5 for day 3 for CD 8^+^ cells).

Figure 7 shows the proportion of CCR7^–^ CD45RA^+^ terminally differentiated CD4^+^ and CD8^+^ T cells. There is a tendency towards decline over time. However, changes are not statistically significant. Additionally, analysis of CCR7^+^ CD45RA^+^ naïve, CCR7^+^ CD45^–^ central memory and CCR7–CD45RA–effector memory was performed in both T cell subtypes with no differences among the different Zn-NP concentrations or between duration of exposure (data not shown).

## 4. Discussion

The effect of nanoparticle (NP) exposure on the human immune system has not yet been adequately investigated. The aim of this study was to obtain a broad overview of the immune modulating potential of ZnO-NP in human lymphocytes from peripheral blood in vitro. The particles used for these experiments did not induce any significant alterations in cell activation markers of lymphocytes, and the composition of T cell subpopulations remained stable. The use of nanoparticles for the production of consumer products has increased over many years, and a variety of different routes exist for a direct entry of NP into the human organism. Considering the most common forms of contact occurring in everyday life, inhalation, dermal exposure and ingestion are among the most relevant. Reports of translocation and accumulation of NP after internalization into the body have been previously described in the literature [18,19]. Bakand and Hayes (2016) illustrated the bio-distribution of NP within the organism, including their uptake into cells of the blood system such as in thrombocytes, monocytes and endothelial cells [20]. Pagano et al. (2019) highlighted the potential effects of various materials on the health of individuals as well, indicating that contact-induced allergies, pulmonary diseases like asthma or carcinogenic effects are possible [21]. In addition to the inevitable exposure of humans through consumer goods, attention should also be paid to the growing use of NP in medicine. NP-based drug delivery systems are being extensively studied for their promising potential in targeted therapies, including anti-cancer treatment. Immunotherapy is increasingly gaining in importance in the context of modern tumor therapy by attempting to boost the intrinsic antitumor activity of the immune system. This can be achieved by inducing the activity of CD8^+^ cytotoxic T lymphocytes by specific antigen presentation in combination with MHC-I molecules [22]. In an activated state, these cells secrete cytokines which further facilitate antigen presentation and mediate anti-cancer effects [23]. Another approach is the use of bispecific antibodies that can direct T effector cells to destroy cancer cells [24]. Furthermore, the downregulation of regulatory T cells as major components of the immune-suppressive tumor microenvironment can attenuate tumor progression [25]. Yet, similar to conventional cancer therapy, there are often obstacles to a broad application of immunotherapy, such as bioavailability and in vivo stability or off-target cytotoxicity. Nonetheless, the use of NP seems promising for enhancing therapeutic efficacy by directly delivering immune-stimulating substances to the site of interest like leucocytes and lymphoid organs. In addition, NP with innately immunogenic properties can be specifically designed to further increase the immune response [26]. However, a precise knowledge of the immune modulating potential of NP in the human organism is not only critical for its potential use in drug therapy, but also to ensure consumer protection in everyday life. 

The exposure times and NP concentrations were chosen within a non-cytotoxic range to ensure that none of the effects were due to cytotoxicity. The incubation for three consecutive days without change of cell culture medium was just tolerable with respect to the supply of lymphocytes with stimulants necessary for survival. Immunomodulation in terms of T cell activation or suppression was evaluated by the flow cytometric detection of distinct surface markers. The proportion of CD4^+^ and CD8^+^ T cells was unaffected by the duration or dose of added NP to the culture. Furthermore, there was no quantifiable significant change in cell activation markers. A decrease in cell activation over time can be explained by the lack of stimulating factors in the cytokine-free culture medium. Greulich et al. (2011) described a significant increase in the early cell activation marker CD69 on primary monocytes after exposure to silver nanoparticles, but not on T cells. One explanation for this observation is the different susceptibility of cells to NP-uptake and intracellular accumulation [27]. Accordingly, Hanley et al. (2009) observed a greater resistance of naïve T cells to ZnO-NP-associated toxicity compared to memory T cells, indicating that the toxic potential of NP is also dependent on the activation status of the cells. Moreover, ZnO-NP induced a significant release of the pro-inflammatory cytokines Interleukin (IL)-12, Interferon-γ (IFN-γ), and Tumor Necrosis Factor-α (TNF-α) in human primary immune cells at concentrations below those causing relevant cell death [28]. The ZnO-NP used in the present study did not alter the appearance of T cell subtypes nor induce T cell differentiation. Overall, NP seem to be immunologically inert in the concentrations tested. 

However, it must be considered that the biological activity of NP is always largely dependent on their physicochemical properties [29]. Size, shape, surface charge, and agglomeration tendencies determine the solubility, bio-distribution and cellular uptake of NP [30,31]. Therefore, a very simple model was used in this study to overcome the lack of being able to simulate the precise processes within the framework of an immune response. For instance, there is evidence that NP interfere with the immune system via Toll-like receptors, which are expressed on antigen-presenting cells (APC) [32]. In the present study, the exposition of lymphocytes to NP took place directly in cell culture medium. It seems that this simple form of contact is not a sufficient stimulus to cause an immunoreaction. To achieve a predictable modification of the immune system, NP can interfere with different components involved in the immune response.

Several studies in which an enhanced activation of CD8^+^ lymphocytes by APC that were specifically targeted with antigen-loaded NP have been described [33,34]. To induce either immune tolerance or stimulation, precise targeting is essential since activation of different APC subpopulations leads to strongly diverse effects. Cruz et al. (2014) described CD8^+^ T cell proliferation and IL release by targeting CD40, DEC205, or CD11c with antigen-loaded NP [35]. Under other conditions, activation of MARCO^+^ macrophages in the liver and spleen induced immune tolerance [36]. Moreover, it is possible to generate nanoparticles which can function as artificial APC to mediate T cell activation. For this purpose, NP must have certain properties: a peptide-MHC complex to achieve interaction with the T cell receptor (TCR), co-stimulatory molecules like the anti-CD28 monoclonal antibody to supply the necessary co-stimulatory signals to T cells, and cytokines, which are essential for T cell expansion and differentiation [37]. 

On the other hand, NP can also trigger an unwanted immunological reaction. NM can be recognized as foreign particles by immune cells, which initiate an inflammatory reaction in order to eliminate them. This involves the activation of helper T cells, neutrophils, and macrophages discharging cytokines, like TNF-α and interleukins (IL-1β, IL-6, IL-12, IL-18) [38]. This pro-inflammatory milieu may lead to serious consequences like systemic inflammation [39]. Mitchell et al. (2009) described immunosuppression of inhaled multi-walled carbon nanotubes in mice. There was no change in the lymphocyte subpopulations, but a decrease in T cell-dependent antibody response to antigen challenge [40]. In the present study, there was no significant change measurable in the activation profile or the distribution of T cell subpopulations. A considerable alteration in cytokine production seems therefore unlikely and has not been specifically investigated thus far. One explanation for the lacking alterations in T cell response might be a deficient exposure time of the cells to NP. Presumably, the continuance of particles in the body after internalization would last much longer. However, an imitation of the physiological conditions in vitro is complex. Due to the cell culture conditions, cell activation markers declined over time in this study. To maintain a stable activation status over a long-time exposure, stimulating factors like IL-2 have to be added to the test solutions, which might also have an influence on the results. The setting of this study was designed to evaluate a wide variety of immunological markers, and a suitable tool was applied by using flow cytometry. In addition, a possible systematic bias of the measurements based on fluorescence due to the intrinsic features of NP must also be considered, although adequate washing steps help to minimize interference in this regard [41]. Furthermore, the design of the study may implicate certain limitations. First, primary cells were used for the experiments, which could suggest that there might be inter-individual characteristics that may potentially influence the results. Moreover, a basic approach was chosen to investigate the initial question of immune modulation through NP exposure. The NP were simply added to the cell culture medium. The effective exposition time and dose of the singular cell is dependent on various factors like particle sedimentation, and are therefore difficult to estimate. As already stated, the short exposure time of only 3 days limits the conclusiveness of the study, and these variables taken overall complicate a comprehensible transference of the measured results to a living organism.

In summary, ZnO-NP used in this study did not appear to induce any immune modulation in human lymphocytes in the tested concentrations. Further studies should focus on a physiological simulation of the interaction of components involved in the immune response. Moreover, the influence of different particles and culture settings should also be evaluated. The performance of confocal imaging or transmission electron microscopy to investigate particle uptake and cellular responses at different time points of incubation with NP would be a challenging but interesting approach to further improve knowledge of the immune modulating potential of NP.

## Figures and Tables

**Table 1:** Results of Annexin-V/Propidiumiodide flow cytometry. Lymphocytes were treated with ZnO-NP between 0.1 and 100 µg/mL for 24 h. Untreated cells served as negative control. Percentages of viable, apoptotic and necrotic cells are illustrated.

	Viable (%)	Apoptotic (%)	Necrotic (%)
negative control	94.2	5.6	0.2
0.1 µg/mL	94.2	5.7	0.1
0.5 µg/mL	95.0	4.09	0.2
1 µg/mL	92.7	6.7	0.4
10 µg/mL	93.7	5.9	0.4
25 µg/mL	78.4	16.2	5.1
50 µg/mL	68.6	20.8	10.3
75 µg/mL	69.1	20.6	10.0
100 µg/mL	63.8	21.6	13.9

**Table 2:** Variation in CD4^+^ subpopulations after incubation with ZnO-NP in different concentrations for 24 h. None of the NP concentrations caused a statistically significant alteration compared to the negative control. Data represent the mean +/− standard deviation (n = 9).

Day 1	Naïve (%)	Memory (%)	rTreg (%)	aTreg (%)	FoxP3^low^ (%)
negative control	51.93 ± 21.41	42.28 ± 0.68	2.20 ± 1.24	1.12 ± 1.10	2.76 ± 1.20
0.1 µg/mL	49.56 ± 22.93	45.68 ± 23.78	1.72 ± 1.73	1.06 ± 1.21	2.21 ± 1.33
0.5 µg/mL	56.07 ± 19.56	39.21 ± 19.88	2.07± 2.27	0.89 ± 0.76	2.17 ± 1.38
1 µg/mL	50.58 ± 21.90	44.60 ± 23.11	1.72 ± 1.42	0.86 ± 0.76	2.38 ± 1.52
10 µg/mL	54.30 ± 19.07	41.66 ± 19.12	1.44 ± 1.40	0.47 ± 0.43	2.48 ± 1.52
positive control	57.97 ± 15.45	26.50 ± 15.15	4.95 ± 2.39	3.88 ± 2.12	4.73 ± 3.30

**Table 3:** Variation in CD4^+^ subpopulations after incubation with ZnO-NP in different concentrations for 48 h. There were no significant differences in the statistical evaluation. Data represent the mean +/− standard deviation (n = 9).

Day 2	Naïve (%)	Memory (%)	rTreg (%)	aTreg (%)	FoxP3^low^ (%)
negative control	52.71 ± 19.87	42.28 ± 20.07	1.60 ± 0.95	0.88 ± 1.00	2.68 ± 1.31
0.1 µg/mL	46.84 ± 22.93	48.40 ± 23.16	1.59 ± 1.46	0.89 ± 0.82	2.63 ± 1.14
0.5 µg/mL	49.82 ± 19.50	46.19 ± 20.48	1.43 ± 1.62	0.79 ± 0.77	2.17 ± 1.30
1 µg/mL	47.96 ± 21.09	48.44 ± 21.62	1.18 ± 0.90	0.72 ± 0.69	2.28 ± 1.36
10 µg/mL	49.44 ± 22.72	46.45 ± 22.36	1.54 ± 1.11	0.48 ± 0.52	2.44 ± 1.20
positive control	53.47 ± 12.47	19.00 ± 9.025	9.04 ± 3.16	7.11 ± 5.40	4.50 ± 3.22

**Table 4:** Variation in CD4^+^ subpopulations after incubation with ZnO-NP in different concentrations for 72 h. There were no significant differences between treated cells and the negative control. Data represent the mean +/− standard deviation (n = 8).

Day 3	Naïve (%)	Memory (%)	rTreg (%)	aTreg (%)	FoxP3^low^ (%)
negative control	48.93 ± 10.28	47.18 ± 10.00	1.11 ± 0.68	0.69 ± 0.63	2.61 ± 0.94
0.1 µg/mL	42.10 ± 13.62	54.66 ± 14.12	0.99 ± 0.98	0.54 ± 0.52	2.09 ± 0.51
0.5 µg/mL	38.94 ± 20.41	57.15 ± 20.60	1.14 ± 1.14	0.69 ± 0.60	2.43 ± 0.51
1 µg/mL	47.05 ± 15.36	50.08 ± 16.43	1.25 ± 0.92	0.47 ± 0.61	1.66 ± 0.76
10 µg/mL	49.53 ± 12.57	46.56 ± 11.58	2.29 ± 3.67	0.35 ± 0.36	1.72 ± 1.08
positive control	43.35 ± 12.94	20.69 ± 7.53	10.74 ± 8.74	9.55 ± 3.89	6.09 ± 3.95
